# Longitudinal associations between changes in higher-level competence and sleep status among community-dwelling older adults in Japan

**DOI:** 10.3389/fpubh.2026.1843833

**Published:** 2026-05-28

**Authors:** Ruifeng Zhao, Shuanghong Li, Haotian Gao, Yixin Sun, Lujiao Huang, Jinrui Zhang, Meiling Qian, Akihiro Kakuda, Yuko Sawada, Tokie Anme

**Affiliations:** 1 Doctoral Program in Medical Sciences, Graduate School of Comprehensive Human Sciences, University of Tsukuba, Tsukuba, Japan; 2 Department of Physical Therapy, Morinomiya University of Medical Sciences, Osaka, Japan; 3 Faculty of Medicine, University of Tsukuba, Tsukuba, Japan

**Keywords:** community-dwelling older adults, higher-level competence, longitudinal study, non-restorative sleep, sleep duration

## Abstract

**Introduction:**

Sleep health is an important component of healthy aging, yet longitudinal evidence on whether changes in higher-level competence are associated with subsequent sleep outcomes remains limited among community-dwelling older adults in Japan. This study examined the longitudinal association between changes in higher-level competence and later sleep status, including short sleep duration and non-restorative sleep, over a 6-year period.

**Methods:**

Data were derived from the Community Empowerment and Care for Wellbeing and Healthy Longevity study conducted from 2017 to 2023 in a suburban Japanese community. Among 1,161 individuals surveyed at baseline, 450 adults aged 65 years and older who had no short sleep duration or non-restorative sleep at baseline and completed follow-up in 2023 were included in the final analysis. Higher-level competence was assessed using the Tokyo Metropolitan Institute of Gerontology Index of Competence, and change scores from 2017 to 2023 were categorized as decline, stable, or improvement. Sleep outcomes at follow-up were defined as incident short sleep duration of less than 6 h per day and non-restorative sleep based on self-report. Multivariable logistic regression analyses were performed, with crude estimates and covariate-adjusted estimates reported separately.

**Results:**

Participants with a decline in higher-level competence had higher odds of short sleep duration (odds ratio, 2.51; 95% confidence interval, 1.33–4.75) and non-restorative sleep (odds ratio, 1.78; 95% confidence interval, 1.11–2.86) compared with those with stable competence, whereas improvement in higher-level competence was not significantly associated with either outcome. In subdimension analyses, decline in social role functioning was associated with short sleep duration, while declines in instrumental activities of daily living and social role functioning were associated with non-restorative sleep.

**Discussion:**

These findings suggest that deterioration in higher-level competence, particularly social role functioning, is longitudinally associated with poorer sleep status among older adults. The results should be interpreted as associations rather than causal effects, but they indicate that functional independence and social engagement may be relevant components of sleep health promotion in later life.

## 1 Introduction

Sleep problems are highly prevalent among older adults worldwide. A meta-analysis of community-dwelling older adults reported a pooled prevalence of 30.5% for sleep disturbances (1), indicating a substantial global burden in later life. In Japan, sleep-related problems are also common among older adults. A large cohort study of community-dwelling older Japanese adults found a 48.7% prevalence of sleep disturbance (2). These findings highlight the importance of identifying modifiable determinants of sleep health in older populations, particularly in Japan. Previous studies have shown that insomnia in older people tends to become chronic (3) and that sleep disorders may increase the risk of psychiatric problems such as depression (4) and cognitive decline (5). In Japan, the *Sleep Guidelines for Health Promotion 2023* clearly state that the national health goals in the sleep domain include increasing the number of people who feel rested after sleep and those who obtain sufficient sleep (6). Therefore, identifying the determinants of sleep health in older adults is of great public health importance.

Higher-level competence refers to the advanced functional abilities of older adults that extend beyond basic activities of daily living (ADL) and reflect an individual's ability for independent living, cognitive functioning, and social participation (7). This concept is typically conceptualized as comprising three major domains: instrumental activities of daily living (IADL), intellectual competence, and social role functioning. IADL includes practical abilities required for independent community living, such as shopping, managing finances, and using transportation (8). Intellectual competence refers to the ability to obtain, understand, and process information (9), whereas social role functioning reflects engagement in social relationships, interaction with the community, and performance of meaningful social roles (10).

The concept of higher-level competence is closely related to the broader discussion of “functioning” in later life. The International Classification of Functioning, Disability, and Health (ICF), adopted by the World Health Organization in 2001 conceptualizes functioning as encompassing all aspects of human life (11). However, when focusing on independence in daily life among older adults, functional capacity provides a more operational and context-specific construct. Lawton proposed a hierarchical model of competence in older adults and argued that independence in later life is supported by multiple levels of ability (12). Within this framework, higher-level functional abilities including instrumental self-maintenance, intellectual activity, and social role functioning form a key theoretical foundation for understanding independent living in old age.

In Japan, higher-level competence is commonly assessed using the Tokyo Metropolitan Institute of Gerontology Index of Competence. The TMIG-IC is a well-established and internationally recognized instrument with confirmed reliability and validity (13), and it has been widely used in studies of older adults both in Japan and internationally (14). Because it captures the three major domains of IADL, intellectual competence, and social role functioning, this index enables comprehensive assessment of how multidimensional functional capacity relates to health-related outcomes in older adults (15).

Higher competence levels may be particularly relevant to sleep health later in life. Intellectual competence is closely associated with cognitive function (16), which is a key determinant of sleep quality in older adults (17). Maintaining intellectual activity may help delay cognitive decline, reduce insomnia related to cognitive impairment, and enhance the ability to cope with anxiety and stress, thereby contributing to better sleep (18, 19). Preserved IADL ability may also support better sleep by promoting physical activity and functional independence, both of which are associated with more stable and restorative sleep (20, 21). In addition, social functioning may influence sleep through psychosocial pathways (22). Older adults who actively participate in social relationships and community activities tend to experience greater emotional stability, lower levels of loneliness, and fewer depressive symptoms (23), all of which are closely associated with sleep quality (24). Furthermore, social participation may also increase daytime activity, help regulate circadian rhythms, and facilitate faster sleep onset and deeper sleep at night (25).

Given the multidimensional nature of higher-level competence (26) and its potential influence on the physical, cognitive, and psychosocial pathways related to sleep, examining its longitudinal association with sleep outcomes may help identify important functional determinants of healthy aging. Although higher-level competence has been extensively studied in relation to disability, mortality, and quality of life (27), longitudinal evidence examining whether changes in higher-level competence are associated with subsequent sleep outcomes remains limited, particularly among community-dwelling older adults in Japan.

Therefore, this study aimed to examine the longitudinal association between changes in higher-level competence and subsequent sleep status, including short sleep duration and non-restorative sleep, among community-dwelling older adults in Japan.

## 2 Methods

### 2.1 Study design and setting

This was a six-year longitudinal analysis conducted from 2017 to 2023 as part of the Community Empowerment and Care for Wellbeing and Healthy Longevity (CEC) study. The CEC study is an ongoing community-based cohort study that began in 1991 in a suburban area of Japan, with self-administered questionnaire surveys conducted every three years.

The study area was a typical suburban Japanese community with a population of approximately 5,000 residents, and all residents were invited to participate in the survey.

### 2.2 Participants

The participants in this analysis were community-dwelling older adults aged 65 years or older at baseline in 2017. To examine incident poor sleep status during follow-up, eligible participants were required to be free from both short sleep duration and non-restorative sleep at baseline. Inclusion criteria were as follows:

1. aged ≥65 years at baseline;
2. having good sleep status at baseline, defined as sleep duration ≥6 h per day and the absence of non-restorative sleep;
3. having complete baseline data as well as completed follow-up in 2023.

The exclusion criteria included participants:

1. with missing baseline data;
2. with poor sleep status at baseline, defined as short sleep duration (< 6 h per day) and/or non-restorative sleep;
3. those who had lost independent living ability, required long-term or intensive care, or had severe physical impairments precluding participation;

According to the participant flow, 1,161 individuals were surveyed in 2017. After excluding 97 individuals with incomplete baseline data, 207 individuals with poor sleep status at baseline, and 104 individuals who required care because of loss of independence, 753 participants were eligible for follow-up.

**Figure 1:**
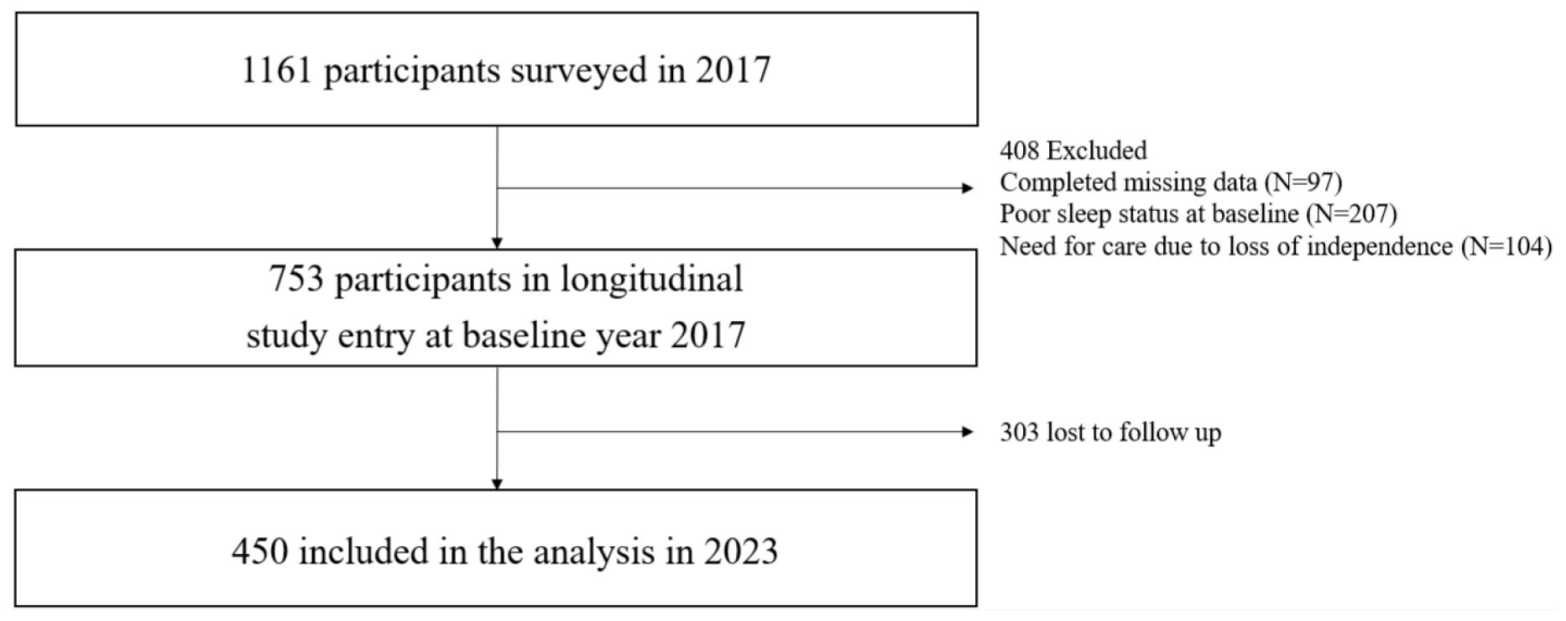
Flowchart of participant selection.

Of these, 303 participants were lost to follow-up, leaving a final analytical sample of 450 participants (Figure 1).

### 2.3 Measurements

#### 2.3.1 Sleep outcomes

Sleep status was assessed using two indicators based on the Sleep Guidelines for Health Promotion 2023 in Japan: sleep duration and subjective sleep restoration (6).

Sleep duration: Participants self-reported their average daily sleep duration. Sleep duration < 6 h per day was defined as short sleep duration.

Sleep restoration: Subjective sleep restoration was assessed using the dichotomous question: “Do you feel adequately rested after sleep?” Participants who responded “No” were classified as having non-restorative sleep.

#### 2.3.2 Higher-level competence

The TMIG-IC was developed by the Tokyo Metropolitan Institute of Gerontology to assess higher-level functional capacity among community-dwelling older adults. It is used to assess the functional abilities of older adults, specifically measuring their autonomy and social participation in daily life. It is primarily used to study and assess the health status of older adults, focusing on their ability to live independently. In Japan, it is regarded as one of the key tools in the field of geriatric health and care, as it provides valuable insights into autonomy and social participation in daily life (28). It is a 13-item scale designed to assess advanced functional capacity in older adults. In the initial pool, on the basis of face validity, items 1–5 had been classified as **Instrumental Activities of Daily Living** (5 points), items 6–9 as **Intellectual Competence** (4 points), and items 10–13 as **Social Role Functioning** (4 points) (29). The response to each item was designed simply as ‘yes' (able to do) or ‘no' (unable), and scored 1 for ‘yes' and 0 for ‘no'. The total score was designed as the sum total of 13 items, or the number of items answered with ‘yes', in order that a higher score (maximum 13 points) could indicate higher competence in older adults. In the course of its development, the reliability was examined. The results showed a reliability coefficient (α) of 0.913 based on Internal Consistency, a correlation coefficient of 0.765 between Inter-Rater (self ratings-family ratings) and a correlation coefficient of 0.859 between Test-Retest, which were all high (13, 30).

Changes in higher-level competence were evaluated by calculating the difference in the total TMIG-IC score between 2017 and 2023 (2023 score minus 2017 score). Because only two measurement points were used in the present analysis, the exposure was treated as a change category rather than as a latent trajectory. Participants were categorized into three groups: decline (score decrease, difference < 0), stable (difference = 0), and improvement (score increase, difference > 0). The stable group was used as the reference category in the logistic regression analyses.

#### 2.3.3 Covariates

The following baseline covariates were considered because of their theoretical relevance to both functional capacity and sleep health: age, sex, exercise habits, body mass index (BMI), smoking status, alcohol consumption, and history of disease. History of disease was defined as hospitalization for more than two weeks during the past year.

### 2.4 Statistical analysis

All statistical analyses were performed using SPSS version 26.0. Descriptive statistics were used to summarize participant characteristics at baseline. Chi-square tests and Mann–Whitney U tests were used for descriptive bivariate comparisons between sleep outcomes and participant characteristics, including TMIG-IC change categories and covariates. These analyses were used to identify variables potentially related to the outcomes and to describe the distribution of baseline characteristics, but causal inference was not based on bivariate significance alone. To assess potential attrition bias, baseline characteristics were compared between the total valid baseline sample and the participants included in the follow-up analysis.

Multivariable logistic regression analyses were performed to estimate the association between changes in higher-level competence and each sleep outcome. Separate models were constructed for incident short sleep duration and non-restorative sleep. Crude models and adjusted models were reported separately to improve transparency. Considering the number of outcome events and the need to avoid overfitting, the adjusted models used a parsimonious outcome-specific covariate set based on baseline characteristics associated with each sleep outcome in bivariate analyses. For short sleep duration, the adjusted model included age, sex, and BMI. For non-restorative sleep, the adjusted model included alcohol consumption, history of disease, and exercise habits. Odds ratios (ORs) and 95% confidence intervals (CIs) were calculated. To evaluate the robustness of the main findings, sensitivity analyses were conducted using fully adjusted logistic regression models in which age, sex, BMI, smoking status, alcohol consumption, exercise habits, and disease history were entered simultaneously, regardless of statistical significance in the univariate analyses.

### 2.5 Ethical considerations

This study was approved by the Ethics Committee of the University of Tsukuba, Japan (approval number: 1331-7). All procedures were conducted in accordance with the Declaration of Helsinki and relevant ethical guidelines. Informed consent was obtained from all participants prior to participation.

## 3 Results

### 3.1 Participant characteristics

Table 1 shows the baseline characteristics of the 450 participants included in the analysis, with mean baseline age of 73.31 ± 6.53 years. Women accounted for 54.2% of the sample, while men accounted for 45.8%. Regarding health-related behaviors, 35.6% of the participants reported smoking, 64.0% reported alcohol consumption, and 56.7% reported engaging in regular exercise. In addition, 78.2% of participants had a history of disease. For BMI, the largest proportion of participants fell within the 18.5–24 kg/m^2^ category (53.1%), followed by those with BMI ≥24 kg/m^2^ (33.6%) and those with BMI < 18.5 kg/m^2^ (13.3%).

**Table 1:** Demographic characteristics of participants (*N* = 450).

Variables	Categories	*n*	%	mean	SD	median	Q25–Q75
Age (years)				73.31	6.53		
Sex	Male	206	45.8				
	Female	244	54.2				
Smoking	Yes	160	35.6				
	No	290	64.4				
Alcohol consumption	Yes	288	64.0				
	No	162	36.0				
Disease	Yes	351	78.0				
	No	99	22.0				
Exercise	Yes	255	56.7				
	No	195	43.3				
BMI	≥24	151	33.6				
	18.5–24	239	53.1				
	< 18.5	60	13.3				
Sleep Duration per day in 2023	≥6 h	379	84.2				
	< 6 h	71	15.8				
Restorative sleep in 2023	Yes	312	69.3				
	No	138	30.7				
TMIG-IC in 2017						12	10–13
TMIG-IC in 2023						12	10–13
Change in TMIG-IC				−0.41	2.42		

Continuous variables are presented as mean and standard deviation (SD) or median and interquartile range (Q25–Q75), as appropriate; categorical variables are presented as number (n) and percentage (%).

With respect to sleep outcomes in 2023, 84.2% of the participants reported a sleep duration of at least six hours per day, whereas 15.8% reported sleeping < 6 h per day. Furthermore, 69.3% reported restorative sleep whereas 30.7% experienced non-restorative sleep.

Regarding higher-level competence, the median TMIG-IC score was 12 (IQR, 10–13) in both 2017 and 2023, indicating a broadly similar overall distribution across the two survey waves. However, the mean change score was −0.41 ± 2.42, suggesting a slight overall decline in higher-level competence during the follow-up period.

### 3.2 Associations between participant characteristics and sleep duration

Bivariate analyses showed that age, sex, BMI, and changes in TMIG-IC were associated with sleep duration. Participants with a decline in TMIG-IC scores were more likely to report short sleep duration at follow-up (Table 2).

**Table 2:** Association between baseline characteristics and sleep duration.

Characteristic	Sleep duration				*χ^2^*	*p*
	Low		Normal			
	*n*	%	*n*	%		
Sex					4.870	0.027
Male	24	33.8	182	48.0		
Female	47	66.2	197	52.0		
Smoking					0.768	0.381
Yes	22	31.0	138	36.4		
No	49	69.0	241	63.6		
Alcohol consumption					1.431	0.232
Yes	41	57.7	247	65.2		
No	30	42.3	132	34.8		
Disease					2.080	0.149
Yes	60	84.5	291	76.8		
No	11	15.5	88	23.2		
Exercise					1.865	0.172
Yes	35	49.3	220	58.0		
No	36	50.7	159	42.0		
BMI					8.509	0.014
≥24	19	26.8	132	34.9		
18.5–24	35	49.3	204	53.8		
< 18.5	17	23.9	43	11.3		
Change in TMIG-IC from 2017 to 2023					7.929	0.019
Decline	35	49.3	129	34.0		
Improvement	18	25.4	92	24.3		
Stable	18	25.4	158	41.7		
Change in instrumental activities of daily living					8.770	0.012
Decline	16	22.5	40	10.6		
Improvement	6	8.5	24	6.3		
Stable	49	69.0	315	83.1		
Change in intellectual competence					17.288	< 0.001
Decline	28	39.4	69	18.2		
Improvement	11	15.5	54	14.2		
Stable	32	45.1	256	67.5		
Change in social role functioning					14.092	< 0.001
Decline	34	47.9	98	25.9		
Improvement	11	15.5	76	20.1		
Stable	26	36.6	205	54.1		
	mean	SD	mean	SD	*Z*	*p*
Age (years)	76.04	7.13	72.80	6.29	−3.648	< 0.001

Low sleep duration was defined as < 6 h per day, and normal sleep duration was defined as ≥6 h per day.

In the multivariable logistic regression analysis, after adjustment for age, sex, and BMI, participants with a decline in TMIG-IC had higher odds of incident short sleep duration than those in the stable group (OR = 2.51, 95% CI: 1.33–4.75). Improvement in TMIG-IC was not significantly associated with incident short sleep duration (Table 3).

**Table 3:** Results of logistic regression analysis for incident short sleep duration according to changes in higher-level competence.

Variable	Category/ comparison	Unadjusted model			Adjusted model		
		OR	95% CI	*p*	OR	95% CI	*p*
Age	Per year	—	—	—	1.07	1.03–1.11	0.001
Sex	Female vs. male	—	—	—	1.75	1.00–3.05	0.050
BMI category	Ordinal	—	—	—	0.67	0.45–1.01	0.053
Change in TMIG-IC	Stable	Ref.	—	—	Ref.	—	—
	Decline	2.38	1.29–4.40	0.006	2.51	1.33–4.75	0.004
	Improvement	1.72	0.85–3.47	0.131	1.65	0.80–3.41	0.172

OR, odds ratio; CI, confidence interval; TMIG-IC, Tokyo Metropolitan Institute of Gerontology Index of Competence. Incident short sleep duration was defined as sleep duration ≥6 h at baseline and < 6 h at follow-up. Adjusted model was adjusted for age, sex, and BMI category. BMI category was entered as an ordinal variable ordered from < 18.5, 18.5–24, to ≥24 kg/m^2^. Stable group was used as the reference category.

### 3.3 Associations between participant characteristics and sleep restoration

The bivariate analyses showed that alcohol consumption, disease history, exercise, and changes in TMIG-IC were associated with sleep restoration. Participants with declining higher-level competence were more likely to experience non-restorative sleep at follow-up (Table 4).

**Table 4:** Association between baseline characteristics and sleep restoration.

Characteristic	Sleep duration				*χ^2^*	*p*
	Low		Normal			
	*n*	%	*n*	%		
Sex					2.167	0.141
Male	56	40.6	150	48.1		
Female	82	59.4	162	51.9		
Smoking					0.052	0.820
Yes	48	34.8	112	35.9		
No	90	65.2	200	64.1		
Alcohol consumption					4.831	0.028
Yes	78	56.5	210	67.3		
No	60	43.5	102	32.7		
Disease					9.305	0.002
Yes	120	87.0	231	74.0		
No	18	13.0	81	26.0		
Exercise					9.834	0.002
Yes	63	45.7	192	61.5		
No	75	54.3	120	38.5		
BMI					2.546	0.280
≥24	41	29.7	110	35.2		
18.5–24	74	53.6	165	52.9		
< 18.5	23	16.7	37	11.9		
Change in TMIG–IC from 2017 to 2023					9.840	0.007
Decline	65	47.1	99	31.7		
Improvement	27	19.6	83	26.6		
Stable	46	33.3	130	41.7		
Change in instrumental activities of daily living					9.620	0.008
Decline	27	19.6	29	9.3		
Improvement	7	5.1	23	7.4		
Stable	104	75.4	260	83.3		
Change in intellectual competence					5.426	0.066
Decline	39	28.3	58	18.6		
Improvement	17	12.3	48	15.4		
Stable	82	59.4	206	66.0		
Change in social role functioning					14.503	< 0.001
Decline	57	41.3	75	24.0		
Improvement	19	13.8	68	21.8		
Stable	62	44.9	169	54.2		
	mean	SD	mean	SD	*Z*	*p*
Age (years)	73.21	6.94	73.36	6.35	−0.564	0.573

Low sleep restoration was defined as non-restorative sleep, and normal sleep restoration was defined as feeling adequately rested after sleep.

In the multivariable logistic regression analysis, after adjustment for alcohol consumption, disease history, and exercise habits, participants with a decline in TMIG-IC had higher odds of non-restorative sleep than those in the stable group (OR = 1.78, 95% CI: 1.11–2.86). Improvement in TMIG-IC was not significantly associated with non-restorative sleep (OR = 0.96, 95% CI: 0.55–1.69) (Table 5).

**Table 5:** Results of logistic regression analysis for non-restorative sleep according to changes in higher-level competence.

Variable	Category/ comparison	Unadjusted model			Adjusted model		
		OR	95% CI	*p*	OR	95% CI	*p*
Alcohol consumption	Yes vs. no	—	—	—	0.64	0.42–0.98	0.038
Disease history	Yes vs. no	—	—	—	2.21	1.26–3.91	0.006
Exercise habits	Yes vs. no	—	—	—	0.56	0.37–0.86	0.007
Change in TMIG-IC	Stable	Ref.	—	—	Ref.	—	—
	Decline	1.86	1.17–2.94	0.008	1.78	1.11–2.86	0.018
	Improvement	0.92	0.53–1.59	0.764	0.96	0.55–1.69	0.884

OR, odds ratio; CI, confidence interval; TMIG-IC, Tokyo Metropolitan Institute of Gerontology Index of Competence. Non-restorative sleep was defined as not feeling adequately rested after sleep. Adjusted model was adjusted for alcohol consumption, disease history, and exercise habits. Stable group was used as the reference category.

### 3.4 Subdimension analyses

In the subdimension analysis, bivariate analyses indicated that changes in Instrumental Activities of Daily Living, Intellectual Competence, and Social Role Functioning were associated with sleep duration (Table 2). In contrast, only changes in Instrumental Activities of Daily Living and Social Role Functioning were associated with sleep restoration (Table 4).

In the multivariable logistic regression analysis for short sleep duration, after adjustment for age, sex, and BMI, decline in Social Role Functioning was associated with higher odds of incident short sleep duration compared with stable Social Role Functioning (OR = 1.97, 95% CI: 1.05–3.70). No significant associations were observed for changes in Instrumental Activities of Daily Living or Intellectual Competence after adjustment for covariates (Table 6).

**Table 6:** Results of logistic regression analysis for incident short sleep duration according to sub-dimensions of change in higher-level competence.

Variable	Category/ comparison	Unadjusted model			Adjusted model		
		OR	95% CI	*p*	OR	95% CI	*p*
Age	Per year	—	—	—	1.05	1.01–1.10	0.012
Sex	Female vs. male	—	—	—	1.52	0.86–2.67	0.146
BMI category	Ordinal	—	—	—	0.71	0.47–1.07	0.105
Change in instrumental activities of daily living	Stable	Ref.	—	—	Ref.	—	—
	Decline	1.62	0.78–3.39	0.198	1.26	0.56–2.83	0.576
	Improvement	1.64	0.61–4.41	0.331	1.22	0.42–3.52	0.718
Change in intellectual competence	Stable	Ref.	—	—	Ref.	—	—
	Decline	2.11	1.09–4.11	0.027	1.93	0.96–3.88	0.066
	Improvement	1.51	0.69–3.31	0.302	1.46	0.66–3.23	0.351
Change in social role functioning	Stable	Ref.	—	—	Ref.	—	—
	Decline	2.04	1.10–3.76	0.023	1.97	1.05–3.70	0.036
	Improvement	1.03	0.47–2.24	0.939	1.07	0.49–2.34	0.874

OR, odds ratio; CI, confidence interval. Incident short sleep duration was defined as sleep duration ≥6 h at baseline and < 6 h at follow-up. In the unadjusted model, the three subdimension change variables were entered simultaneously without covariates. The adjusted model included the three subdimension change variables and was additionally adjusted for age, sex, and BMI category. BMI category was entered as an ordinal variable ordered from < 18.5, 18.5–24, to ≥24 kg/m^2^. Stable groups were used as the reference categories.

In the multivariable logistic regression analysis for non-restorative sleep, after adjustment for alcohol consumption, disease history, and exercise, decline in Instrumental Activities of Daily Living (OR = 1.92, 95% CI: 1.05–3.53) and decline in Social Role Functioning (OR = 1.81, 95% CI: 1.13–2.91) were associated with higher odds of non-restorative sleep than the corresponding stable groups (Table 7).

**Table 7:** Results of logistic regression analysis for non-restorative sleep according to sub-dimensions of change in higher-level competence.

Variable	Category/ comparison	Unadjusted model			Adjusted model		
		OR	95% CI	*p*	OR	95% CI	*p*
Alcohol consumption	Yes vs. no	—	—	—	0.65	0.42–1.00	0.051
Disease history	Yes vs. no	—	—	—	2.22	1.25–3.94	0.006
Exercise habits	Yes vs. no	—	—	—	0.57	0.38–0.88	0.010
Change in instrumental activities of daily living	Stable	Ref.	—	—	Ref.	—	—
	Decline	2.01	1.12–3.61	0.019	1.92	1.05–3.53	0.035
	Improvement	0.87	0.36–2.12	0.759	0.83	0.33–2.06	0.682
Change in social role functioning	Stable	Ref.	—	—	Ref.	—	—
	Decline	1.86	1.17–2.95	0.009	1.81	1.13–2.91	0.014
	Improvement	0.78	0.43–1.42	0.420	0.83	0.46–1.52	0.552

OR, odds ratio; CI, confidence interval. Non-restorative sleep was defined as not feeling adequately rested after sleep. In the unadjusted model, changes in Instrumental Activities of Daily Living and Social Role Functioning were entered simultaneously without covariates. The adjusted model included these subdimension change variables and was additionally adjusted for alcohol consumption, disease history, and exercise habits. Stable groups were used as the reference categories.

### 3.5 Sensitivity analyses

In the fully adjusted sensitivity analyses, decline in total TMIG-IC remained associated with short sleep duration (aOR = 2.39, 95% CI: 1.26–4.53) and non-restorative sleep (aOR = 1.85, 95% CI: 1.14–2.99). In the subdimension models, decline in social role functioning remained associated with both short sleep duration (aOR = 1.95, 95% CI: 1.03–3.69) and non-restorative sleep (aOR = 1.89, 95% CI: 1.14–3.13), while decline in instrumental activities of daily living was associated with non-restorative sleep (aOR = 2.03, 95% CI: 1.03–3.99). These findings were generally consistent with the primary analyses (Supplementary Table S1).

## 4 Discussion

This longitudinal study examined whether changes in higher-level competence were associated with subsequent sleep status among community-dwelling older adults in Japan over a six-year period. The results showed that decline in higher-level competence was associated with higher odds of both incident short sleep duration and non-restorative sleep. In particular, participants with declining TMIG-IC scores had poorer sleep outcomes than those who maintained stable competence. Although previous studies have primarily focused on frailty, disability, or social vulnerability rather than higher-level competence, they consistently indicate that poorer functional status and sleep problems are closely related in later life. For example, a Japanese longitudinal study reported a bidirectional relationship between insomnia and frailty among community-dwelling older adults, suggesting that sleep problems and functional decline may reinforce each other over time (31). The present findings extend this line of evidence by focusing on change in higher-level competence as a multidimensional indicator of functional aging. The fully adjusted sensitivity analyses further supported the robustness of the main findings, suggesting that the observed associations were not solely explained by differences in demographic characteristics, health behaviors, BMI, or disease history.

The observed associations are biologically and socially plausible, but they should not be interpreted as evidence of a causal effect. Higher-level competence reflects a multidimensional functional capacity encompassing cognitive, physical, and social domains of everyday life. Decline in these domains may be linked to sleep through several pathways, including reduced daytime activity, weakened social engagement, emotional distress, and disruption of daily routines. This interpretation is broadly consistent with previous studies showing that sleep and functional ability are closely linked, although many earlier studies have examined the reverse direction of this association. For instance, longitudinal evidence from China showed that both short and long sleep durations predicted incident IADL disability and that cognition partially mediated this relationship (32). Taken together, these findings suggest that the relationship between sleep and functional capacity may be reciprocal rather than unidirectional.

With respect to domain-specific findings, the three TMIG-IC domains may relate to sleep through partly different mechanisms. Intellectual competence is closely related to cognitive health. Reduced cognitive engagement may be associated with poorer emotional regulation, vulnerability to stress, and cognitive decline, all of which may increase the likelihood of sleep problems. IADL reflects the ability to maintain active and independent daily living. Older adults with reduced IADL may have lower daytime activity levels and poorer physical functioning, which may weaken sleep regulation and reduce perceived sleep restoration. This explanation is consistent with previous findings showing that physical activity and functional independence are important correlates of sleep quality in older adults. In a Japanese longitudinal study, older adults with high physical activity and strong social relationships had a significantly lower risk of sleep disorders than those with low activity and weak social relationships (33). Social role functioning may influence sleep through psychosocial and behavioral mechanisms. Older adults who maintain social engagement are less likely to experience loneliness and depressive symptoms, and may have more structured daily routines, both of which are beneficial for sleep quality and circadian rhythm regulation.

Among the three TMIG-IC domains, social role functioning showed a particularly consistent association with sleep outcomes. This finding suggests that social role functioning may be one of the most relevant aspects of higher-level competence for sleep health in later life. Participation in meaningful social roles may help older adults maintain emotional stability, a sense of belonging, and regular daytime activities, all of which may support healthier sleep patterns. This finding is in line with previous studies that emphasized the importance of social relationships for sleep in older adults. A cross-sectional Japanese study found that social frailty was associated with poorer subjective sleep quality, particularly among individuals who did not engage in daily social interactions (34). Similarly, an earlier study reported that older adults with social participation had better sleep outcomes as measured by actigraphy. However, that study did not find strong evidence that increasing social participation improved sleep over a five-year period, suggesting that the relationship may be influenced by self-selection or may operate over shorter or more dynamic time frames (35). In this context, our findings add longitudinal evidence that deterioration in social role functioning is associated with later poor sleep status, while causal direction should still be interpreted cautiously.

Notably, although changes in Instrumental Activities of Daily Living, Intellectual Competence, and Social Role Functioning were all associated with sleep duration in the bivariate analysis, only change in Social Role Functioning remained significant after adjustment for covariates. This finding suggests that social role functioning may have a more independent association with sleep duration in older adults, whereas the associations of the other sub-dimensions may be partly explained by shared variance or confounding factors. One possible explanation is that social role functioning more directly reflects the extent to which older adults maintain interpersonal engagement, meaningful social roles, and structured daily activities, all of which may help regulate daytime activity patterns, emotional stability, and circadian rhythm, thereby supporting healthier sleep duration (35, 36).

In contrast, change in Intellectual Competence was not significantly associated with non-restorative sleep. One possible explanation is that non-restorative sleep may be more strongly related to functional independence and social engagement than to intellectual competence alone. Instrumental Activities of Daily Living and Social Role Functioning may more directly reflect daytime activity patterns, interpersonal interaction, and role participation, all of which are closely related to subjective sleep restoration (37). By comparison, the association between Intellectual Competence and non-restorative sleep may be weaker, more indirect (21), or partly shared with other dimensions of higher-level competence, making its independent association more difficult to detect. Limited statistical power in the subdimension analysis may also have contributed to the lack of significance.

The present study had several strengths. First, it used longitudinal data over a 6-year follow-up period, allowing temporal ordering between changes in competence and later sleep outcomes to be examined. Second, it focused on community-dwelling older adults, a population that is highly relevant to preventive public health strategies. Third, the study used the TMIG-IC, a well-established measure of higher-level competence that captures multiple domains of functional aging. Fourth, crude and adjusted models were presented separately, and the participant flow and attrition comparison were reported to improve transparency. Compared with previous studies that focused separately on frailty, disability, or social participation, the present study provides a more integrated functional perspective by examining higher-level competence as a multidimensional construct.

However, several limitations should be acknowledged. First, sleep outcomes were assessed by self-report rather than by objective measures, which may have introduced reporting bias. This may partly explain discrepancies between our findings and those of previous studies using objective assessments such as actigraphy (35). Second, participants were recruited from a single suburban Japanese community, which may limit the generalizability of the findings. Third, the study sample was restricted to relatively healthier and functionally independent older adults, as individuals who had lost independent living ability, required long-term or intensive care, or had severe physical impairments precluding participation were not included in the analysis. Therefore, the findings may not be fully generalizable to older adults with greater functional impairment or care needs. Fourth, a substantial number of participants were lost to follow-up during the six-year observation period. Although some attrition was unavoidable in this long-term community-based study because of advanced age, declining health, hospitalization, death, relocation, loss of contact, or reduced willingness to participate, such attrition may still have introduced selection bias. However, a comparison of baseline characteristics between the total valid sample and the follow-up sample showed no substantial differences in the main characteristics (Supplementary Table S2). Fifth, the adjusted models used parsimonious outcome-specific covariate sets to reduce overfitting given the number of outcome events. Although this approach improved model stability, it may not have fully controlled for all theoretically relevant confounders, and residual confounding may remain. Finally, short sleep was defined only as less than 6 h, although long sleep duration may also be associated with adverse health outcomes in older adults. As a result, the present study may not have fully captured the potentially U-shaped relationship between sleep duration and health, and some participants with excessively long sleep may have been classified as having normal sleep (38, 39). Future studies should assess both short and long sleep duration for a more comprehensive evaluation of sleep health.

Despite these limitations, the findings suggest that changes in higher-level competence, especially social role functioning, are longitudinally associated with sleep deterioration among community-dwelling older adults. These results do not establish causality, but they indicate that preserving independence, promoting social participation, and supporting cognitive activity may be relevant components of sleep health promotion in later life. In light of prior evidence on frailty, social vulnerability, and sleep, sleep promotion strategies for older adults may benefit from incorporating functional and social components rather than focusing solely on biomedical or behavioral sleep interventions (40, 41).

## 5 Conclusions

In this six-year longitudinal study of community-dwelling older adults in Japan, decline in higher-level competence was associated with higher odds of incident short sleep duration and non-restorative sleep. Among the sub-dimensions of higher-level competence, social role functioning showed a particularly consistent association with sleep outcomes. These findings suggest that maintaining higher-level competence may be relevant to sleep health in later life. From a public health perspective, approaches that support functional independence and social engagement may contribute to better sleep outcomes among older adults, although further studies using objective sleep measures and more comprehensive adjustment for confounding are needed.

## Data Availability

The data analyzed in this study are not publicly available because they contain information from a community-based cohort and are subject to privacy and ethical restrictions. Requests to access the datasets should be directed to the corresponding author, Tokie Anme, at anmet@md.tsukuba.ac.jp, and will be considered upon reasonable request and with permission from the relevant local government and ethics committee where applicable.

## References

[1] Bao Y-P Han Y Ma J Wang R-J Shi L Wang T-Y . Cooccurrence and bidirectional prediction of sleep disturbances and depression in older adults: meta-analysis and systematic review. Neurosci Biobehav Rev. (2017) 75:257–73. doi: 10.1016/j.neubiorev.2017.01.032 28179129

[2] Watanabe D Yoshida T Watanabe Y Yamada Y Miyachi M Kimura M . Combined use of sleep quality and duration is more closely associated with mortality risk among older adults: a population-based kyoto-kameoka prospective cohort study. J Epidemiol. (2023) 33:591–9. doi: 10.2188/jea.JE20220215 36155361 PMC10635816

[3] Patel D Steinberg J Patel P. Insomnia in the elderly: a review. J Clin Sleep Med. (2018) 14:1017–24. doi: 10.5664/jcsm.7172 29852897 PMC5991956

[4] Nielson SA Kay DB Dzierzewski JM. Sleep and depression in older adults: a narrative review. Curr Psychiatry Rep. (2023) 25:643–58. doi: 10.1007/s11920-023-01455-3 37740851

[5] Chen J Chen X Mao R Fu Y Chen Q Zhang C . Hypertension, sleep quality, depression, and cognitive function in elderly: a cross-sectional study. Front Aging Neurosci. (2023) 15:1051298. doi: 10.3389/fnagi.2023.1051298 36824262 PMC9942596

[6] Ministry Ministry of Health Labour and Welfare. Sleep Guidelines for Health Promotion 2023 (2023). Available online at: https://www.mhlw.go.jp/content/001305530.pdf (Accessed January 01, 2026).

[7] Koyano W Shibata H Nakazato K Haga H Suyama Y. Toward measuring the activity competence of community-dwelling older adults. Soc Gerontol. (1986) 23:35–43.

[8] Pashmdarfard M Azad A. Assessment tools to evaluate activities of daily living (ADL) and instrumental activities of daily living (IADL) in older adults: a systematic review. Med J Islam Repub Iran. (2020) 34:33. doi: 10.47176/mjiri.34.33 32617272 PMC7320974

[9] Fernández I García-Mollá A Oliver A Sansó N Tomás J. The role of social and intellectual activity participation in older adults' cognitive function. Arch Gerontol Geriatr. (2022) 107:104891. doi: 10.1016/j.archger.2022.104891 36521393

[10] Levasseur M Lussier-Therrien M Biron M Raymond É Castonguay J Naud D . Scoping study of definitions of social participation: update and co-construction of an interdisciplinary consensual definition. Age Ageing. (2022) 51:afab215. doi: 10.1093/ageing/afab215 35134843 PMC9383398

[11] Buñuales M Diego G Moreno J. International classification of functioning, disability and health (ICF) 2001. Revista Española de Salud Pública. (2002) 76:271–9. doi: 10.1590/s1135-57272002000400002 12216167

[12] Lawton MP. Assessing the Competence of Older People. In: Kent DP, Kastenbaum R, Sherwood S, editors. Research Planning and Action for the Elderly: The Power and Potential of Social Science. New York, NY: Behavioral Publications (1972). p. 122–43.

[13] Koyano W Shibata H. Cross-validity of the TMIG index of competence: invariance of factor structure and predictive validity. Japanese J Gerontol. (1992) 14:34–42.

[14] Otsuka R Nishita Y Tange C Tomida M Kato Y Nakamoto M . The effect of modifiable healthy practices on higher-level functional capacity decline among Japanese community dwellers. Prev Med Rep. (2016) 5:205–9. doi: 10.1016/j.pmedr.2016.12.022 28070478 PMC5219638

[15] Iwasa H Masui Y Inagaki H Yoshida Y Shimada H Otsuka R . Assessing competence at a higher level among older adults: development of the Japan Science and technology agency index of competence (JST-IC). Aging Clin Exp Res. (2018) 30:383–93. doi: 10.1007/s40520-017-0786-8 28646250

[16] Raimo S Maggi G Ilardi C Cavallo N Torchia V Pilgrom M . The relation between cognitive functioning and activities of daily living in normal aging, mild cognitive impairment, and dementia: a meta-analysis. Neurol Sci. (2024) 45:2427–43. doi: 10.1007/s10072-024-07366-2 38347298

[17] Qin S Leong R Ong J Chee M. Associations between objectively measured sleep parameters and cognition in healthy older adults: a meta-analysis. Sleep Med Rev. (2022) 67:101734. doi: 10.1016/j.smrv.2022.101734 36577339

[18] Zhang N Chen F Wang C Yan P. Incidence of cognitive impairment after hypothetical interventions on depression, nighttime sleep duration, and leisure activity engagement among older Chinese adults: an application of the parametric g-formula. Front Public Health. (2023) 11:1088833. doi: 10.3389/fpubh.2023.1088833 36875389 PMC9975736

[19] Yates L Ziser S Spector A Orrell M. Cognitive leisure activities and future risk of cognitive impairment and dementia: systematic review and meta-analysis. Int Psychogeriatr. (2016) 28:1791–806. doi: 10.1017/S1041610216001137 27502691

[20] Cai D Zeng Y Chen M Zhong Y Quan Y Ye M . Association between sleep duration and disability in activities of daily living among Chinese older adults: a nationwide observational study. Front Public Health. (2025) 13:1580101. doi: 10.3389/fpubh.2025.1580101 40469604 PMC12133472

[21] Lee Y Kong D Lee Y Lin C Liu C Chang Y. Functional disabilities and changes in sleep quality and duration among older adults: results from a longitudinal study in China, 2005–2014. Eur Geriatr Med. (2022) 13:967–75. doi: 10.1007/s41999-022-00619-3 35191012

[22] Gordon A Carrillo B Barnes C. Sleep and social relationships in healthy populations: a systematic review. Sleep Med Rev. (2021) 57:101428. doi: 10.1016/j.smrv.2021.101428 33596514

[23] Liang L. The impact of social participation on the quality of life among older adults in China: a chain mediation analysis of loneliness, depression, and anxiety. Front Public Health. (2024) 12:1473657. doi: 10.3389/fpubh.2024.1473657 39386948 PMC11461257

[24] Wang Y Zheng F Zhang X. The impact of social participation on frailty among older adults: the mediating role of loneliness and sleep quality. Healthcare. (2024) 12:2085. doi: 10.3390/healthcare12202085 39451499 PMC11507455

[25] Park S Zhunis A Constantinides M Aiello L Quercia D Cha M. Social dimensions impact individual sleep quantity and quality. Sci Rep. (2023) 13:9681. doi: 10.1038/s41598-023-36762-5 37322226 PMC10272146

[26] Iwasa H Masui Y Inagaki H Yoshida Y Shimada H Otsuka R . Development of the Japan science and technology agency index of competence to assess functional capacity in older adults. Gerontol Geriatr Med. (2015) 1:2333721415609490. doi: 10.1177/2333721415609490 28138472 PMC5119882

[27] Koyano W Hashimoto M Fukawa T Shibata H Gunji A. Functional capacity of community-dwelling older adults: distribution of scores based on the TMIG Index of Competence. Japanese J Public Health. (1993) 40:468–74. 8347862

[28] Kawai H Imamura K Ejiri M Fujiwara Y Ihara K Hirano H . Aging trajectories of subscales in higher-level functional capacity among community-dwelling older Japanese adults: the Otassha study. Aging Clin Exp Res. (2024) 36:137. doi: 10.1007/s40520-024-02791-x 38904857 PMC11192685

[29] Koyano W. Measurement of competence in the elderly living at home: development of an index of competence. Japanese J Public Health. (1987) 34:109–14.

[30] Koyano W Shibata H Nakazato K Haga H Suyama Y. Measurement of competence: reliability and validity of the TMIG index of competence. Arch Gerontol Geriatr. (1991) 13:103–16. doi: 10.1016/0167-4943(91)90053-S 15374421

[31] Nemoto Y Sato S Kitabatake Y Nakamura M Takeda N Maruo K . Bidirectional relationship between insomnia and frailty in older adults: a 2-year longitudinal study. Arch Gerontol Geriatr. (2021) 97:104519. doi: 10.1016/j.archger.2021.104519 34564037

[32] Liu M Du X Sun Y Zhou A Sun S Wu Y. The mediating role of cognition in the relationship between sleep duration and instrumental activities of daily living disability among middle-aged and older Chinese. Arch Gerontol Geriatr. (2021) 94:104369. doi: 10.1016/j.archger.2021.104369 33556636

[33] Seol J Lee J Nagata K Fujii Y Joho K Tateoka K . Combined effect of daily physical activity and social relationships on sleep disorder among older adults: cross-sectional and longitudinal study based on data from the Kasama study. BMC Geriatr. (2021) 21:623. doi: 10.1186/s12877-021-02589-w 34732144 PMC8565015

[34] Noguchi T Nojima I Inoue-Hirakawa T Sugiura H. Association between social frailty and sleep quality among community-dwelling older adults: a cross-sectional study. Phys Ther Res. (2021) 24:153–62. doi: 10.1298/ptr.E10085 34532211 PMC8419475

[35] Chen J Lauderdale D Waite L. Social participation and older adults' sleep. Soc Sci Med. (2015) 149:164–73. doi: 10.1016/j.socscimed.2015.11.045 26724432 PMC4718773

[36] Peng L Peng Y Huang W. Social participation and insomnia in Chinese older adults with multimorbidity: mediating roles of frailty, anxiety, and depression. BMC Geriatr. (2025) 25:605. doi: 10.1186/s12877-025-06299-5 40781598 PMC12333280

[37] Folorunsho S Abraham O Esiaka D. Social engagement moderates the relationship between cognitive functioning, depressive symptoms, and restless sleep in older black adults. Alzheimers Dementia. (2026) 22:e71078. doi: 10.1002/alz.71078 PMC12796501 41527277

[38] Da Silva A De Mello R Schaan C Fuchs F Redline S Fuchs S. Sleep duration and mortality in the elderly: a systematic review with meta-analysis. BMJ Open. (2016) 6:e008119. doi: 10.1136/bmjopen-2015-008119 PMC4762152 26888725

[39] Jike M Itani O Watanabe N Buysse D Kaneita Y. Long sleep duration and health outcomes: a systematic review, meta-analysis and meta-regression. Sleep Med Rev. (2017) 39:25–36. doi: 10.1016/j.smrv.2017.06.011 28890167

[40] Huang Y Fleury J. Socially-supported sleep in older adults aged 50 and older: a concept analysis. Front Public Health. (2024) 12:1364639. doi: 10.3389/fpubh.2024.1364639 38645458 PMC11027164

[41] Salehi Z Pasha H Hosseini S Kheirkhah F Bijani A. The impact of social support, physical and psychological performance on sleep outcomes in Iranian older adults: a case-control study. BMC Geriatr. (2023) 23:791. doi: 10.1186/s12877-023-04455-3 38041024 PMC10693071

