# Supplementary material for: Longitudinal associations between changes in higher-level competence and sleep status among community-dwelling older adults in Japan
Source: Front Public Health. 2026 May 28;14:1843833. doi: 10.3389/fpubh.2026.1843833 (PMC13253282; doi:10.3389/fpubh.2026.1843833)
Supplement: Supplementary file 1 [file Supplementary_Tables.docx]

**Supplementary Table S1. Sensitivity analysis using fully adjusted logistic regression models**

Model 1. Fully adjusted model for short sleep duration using total TMIG-IC change

| Variable | Category/comparison | aOR | 95% CI | *p* |
| --- | --- | --- | --- | --- |
| Age | Per year | 1.06 | 1.02–1.11 | 0.002 |
| Sex | Female vs male | 2.02 | 0.97–4.21 | 0.061 |
| BMI category | Ordinal | 0.66 | 0.44–0.98 | 0.041 |
| Smoking | Yes vs no | 1.28 | 0.60–2.74 | 0.530 |
| Alcohol consumption | Yes vs no | 1.00 | 0.55–1.80 | 0.988 |
| Exercise | Yes vs no | 0.74 | 0.43–1.28 | 0.281 |
| Disease history | Yes vs no | 1.30 | 0.63–2.66 | 0.477 |
| Change in TMIG-IC | Stable | Ref. | — | — |
|  | Decline | 2.39 | 1.26–4.53 | 0.008 |
|  | Improvement | 1.62 | 0.78–3.36 | 0.192 |

Model 2. Fully adjusted model for non-restorative sleep using total TMIG-IC change

| Variable | Category/comparison | aOR | 95% CI | *p* |
| --- | --- | --- | --- | --- |
| Age | Per year | 0.98 | 0.95–1.01 | 0.199 |
| Sex | Female vs male | 1.45 | 0.82–2.57 | 0.203 |
| BMI category | Ordinal | 0.76 | 0.55–1.05 | 0.092 |
| Smoking | Yes vs no | 1.22 | 0.67–2.21 | 0.517 |
| Alcohol consumption | Yes vs no | 0.67 | 0.42–1.07 | 0.096 |
| Exercise | Yes vs no | 0.55 | 0.35–0.84 | 0.006 |
| Disease history | Yes vs no | 2.38 | 1.34–4.25 | 0.003 |
| Change in TMIG-IC | Stable | Ref. | — | — |
|  | Decline | 1.85 | 1.14–2.99 | 0.012 |
|  | Improvement | 0.95 | 0.54–1.69 | 0.874 |

Model 3. Fully adjusted model for short sleep duration using changes in TMIG-IC subdimensions

| Variable | Category/comparison | aOR | 95% CI | *p* |
| --- | --- | --- | --- | --- |
| Age | Per year | 1.05 | 1.01–1.10 | 0.021 |
| Sex | Female vs male | 1.69 | 0.80–3.58 | 0.168 |
| BMI category | Ordinal | 0.69 | 0.45–1.04 | 0.079 |
| Smoking | Yes vs no | 1.16 | 0.53–2.53 | 0.710 |
| Alcohol consumption | Yes vs no | 1.05 | 0.57–1.93 | 0.865 |
| Exercise | Yes vs no | 0.76 | 0.44–1.32 | 0.334 |
| Disease history | Yes vs no | 1.42 | 0.68–2.94 | 0.348 |
| Change in IADL | Stable | Ref. | — | — |
|  | Decline | 1.19 | 0.52–2.69 | 0.685 |
|  | Improvement | 1.17 | 0.40–3.42 | 0.775 |
| Change in Intellectual Competence | Stable | Ref. | — | — |
|  | Decline | 1.94 | 0.95–3.94 | 0.068 |
|  | Improvement | 1.48 | 0.67–3.29 | 0.332 |
| Change in Social Role Functioning | Stable | Ref. | — | — |
|  | Decline | 1.95 | 1.03–3.69 | 0.040 |
|  | Improvement | 1.08 | 0.49–2.37 | 0.850 |

Model 4. Fully adjusted model for non-restorative sleep using changes in TMIG-IC subdimensions

| Variable | Category/comparison | aOR | 95% CI | *p* |
| --- | --- | --- | --- | --- |
| Age | Per year | 0.97 | 0.93–1.00 | 0.062 |
| Sex | Female vs male | 1.35 | 0.76–2.41 | 0.311 |
| BMI category | Ordinal | 0.80 | 0.57–1.11 | 0.181 |
| Smoking | Yes vs no | 1.20 | 0.65–2.19 | 0.563 |
| Alcohol consumption | Yes vs no | 0.66 | 0.41–1.06 | 0.084 |
| Exercise | Yes vs no | 0.56 | 0.36–0.87 | 0.009 |
| Disease history | Yes vs no | 2.47 | 1.37–4.44 | 0.003 |
| Change in IADL | Stable | Ref. | — | — |
|  | Decline | 2.03 | 1.03–3.99 | 0.040 |
|  | Improvement | 0.98 | 0.37–2.54 | 0.959 |
| Change in Intellectual Competence | Stable | Ref. | — | — |
|  | Decline | 1.01 | 0.56–1.83 | 0.976 |
|  | Improvement | 0.97 | 0.50–1.88 | 0.932 |
| Change in Social Role Functioning | Stable | Ref. | — | — |
|  | Decline | 1.89 | 1.14–3.13 | 0.013 |
|  | Improvement | 0.84 | 0.46–1.55 | 0.578 |

Note. aOR = adjusted odds ratio; CI = confidence interval; TMIG-IC = Tokyo Metropolitan Institute of Gerontology Index of Competence; IADL = Instrumental Activities of Daily Living. All models were adjusted for age, sex, BMI, smoking status, alcohol consumption, exercise habits, and disease history. Stable groups were used as the reference categories for TMIG-IC change variables. Short sleep duration was defined as sleep duration ≥6 hours at baseline but <6 hours at follow-up. Non-restorative sleep was defined as not feeling adequately rested after sleep. BMI category was entered as an ordinal variable.

**Supplementary Table S2. Comparison of baseline characteristics between the follow-up sample (n = 450) and the total valid sample (n = 753)**

| Characteristic | χ²/Z | *p* |
| --- | --- | --- |
| TMIG-IC points in 2017 | 1.291 | 0.167 |
| Age | -1.751 | 0.056 |
| Sex | 0.134 | 0.787 |
| Smoking | 0.218 | 0.698 |
| Alcohol consumption | 0.799 | 0.373 |
| Disease | 2.163 | 0.078 |
| Exercise | 0.121 | 0.745 |
| BMI | 1.526 | 0.102 |
